# Innovative Antimicrobial Fabrics Loaded with Nanocomposites from Chitosan and Black Mulberry Polysaccharide-Mediated Selenium Nanoparticles to Suppress Skin Pathogens

**DOI:** 10.3390/polym17212902

**Published:** 2025-10-30

**Authors:** Mousa Abdullah Alghuthaymi

**Affiliations:** Applied College, Shaqra University, Alquwayiyah 11971, Saudi Arabia; malghuthaymi@su.edu.sa

**Keywords:** antimicrobial textiles, biopolymers, green synthesis, nanoconjugates, pathogenic microbes, skin protection

## Abstract

Skin pathogenic microbes continue to seriously endanger humans, particularly resistant strains. Nanomaterials/composites are promising answers for this. Black mulberry (MB) polysaccharides were employed for biosynthesizing/capping selenium nanoparticles (SeNPs); their conjugations alongside chitosan (Cht) nanoforms were constructed and assessed for skin pathogens’ (*Staphylococcus aureus* bacteria and *Candida albicans* yeast) suppression and destruction. The biosynthesis of SeNPs with MB was verified using FTIR analysis and UV-vis spectroscopy. The nanocomposites were constructed from Cht–MB-SeNPs at concentrations of 2:1 (F1), 1:1 (F2), and 1:2 (F3). The SeNPs had a mean diameter of 46.19 nm, whereas the F-2 nanocomposites had the lowest particle diameter (212.42 nm) compared to F-1 (239.88 nm) and F-3 (266.16 nm) nanocomposites. The F-2 nanocomposites significantly exhibited the strongest antimicrobial efficacy against skin pathogens, with 26.3 and 27.1 mm inhibition zones and 22.5 and 20.0 μg/mL inhibitory concentrations against bacteria and *C. albicans* yeast, respectively. The scanning imaging of microbes exposed to nanocomposite emphasized the severe destruction/lyses of microbial cells within 10 h. Loading of cotton fabrics with nanomaterials, particularly with Cht/MB-SeNP nanocomposites, generated potent durable antimicrobial textiles that could prohibit microbial growth, with inhibition zones of 6.2 mm against *C. albicans* and 3.7 mm against *S. aureus*; the textiles could preserve their antimicrobial actions after two washing cycles. The biogenic construction of Cht/MB-SeNP nanocomposites can provide innovative solutions to manage and control skin pathogens.

## 1. Introduction

Nanotechnology, often called the science of miniature scales, has sparked immense interest among researchers, inventors, and technicians across the globe [1]. Essentially, it involves creating and handling materials at the nanoscale level, where sizes are quantified in nanometers—one-billionth of a meter. This tiny dimension unlocks vast opportunities, allowing precise engineering of substances at the molecular and atomic scales [2]. This field has rapidly emerged as a key catalyst for progress in science and technology, influencing areas like electronics, healthcare, material engineering, power generation, and ecological preservation [3]. It holds the potential to transform sectors, deepen our grasp of physical principles, and tackle some of the world’s most urgent issues [4].

In recent times, metal-based nanoparticles have risen as among the most prominent and rapidly evolving substances in scientific research [5]. Selenium nanoparticles (SeNPs) have attracted considerable focus, and their creation could offer substantial benefits in multiple sectors. Methods for producing SeNPs using plant-based extracts steer clear of toxic substances, rely on affordable and accessible starting materials, and avoid the need for specialized environments [6]. Furthermore, this approach allows for accurate regulation of the nanoparticles’ form, dimensions, and durability [7]. Thanks to their potent antimicrobial effects, ability to shield cells from oxidative stress, low toxicity, high biological performance, and favorable absorption rates, SeNPs outperform other forms of selenium (Se) [8].

SeNPs hold promise as antimicrobial agents in biopolymers for use in food and drug sectors [9]. Traditionally, SeNPs were made through physical or chemical processes, but these often involved harsh chemicals, extreme heat, and low pH levels, yielding particles unfit for food packaging applications [10]. In contrast, eco-friendly synthesis using extracts from plants or microbes has become popular lately [8]. Biologically produced nanoparticles are free from chemical contaminants, rendering them safe, simple, and economical [11].

The mulberry plant (*Morus* spp.) grows extensively around the world, particularly in tropical and moderate climates [12]. Primary growing regions include the Middle East, Eastern Asia, and Southwestern Asia [13]. Common varieties encompass red mulberry (*Morus rubra* Linn.), black mulberry (*Morus nigra* Linn.), and white mulberry (*Morus alba* Linn.) [14]. Polysaccharides from plants are abundant in leaves, fruits, and blooms, possessing bioactive traits [15]. Their antioxidative capabilities are drawing increasing academic interest, following compounds like polyphenols, flavonoids, and tannins [5]. Polysaccharides represent the primary bioactive element in mulberries [16], forming a pectin-like substance isolated and refined from the fruits. Various investigations have demonstrated that mulberry polysaccharides exhibit robust antioxidative effects both inside and outside the body, with links to their structural features and functions [17,18]. Additional research indicates that the strong antioxidative qualities of these polysaccharides contribute to protecting the liver from damage [18]. The overall phenolic and flavonoid levels in *M. nigra* fruits range from 485.5 to 1580 mg Gallic Acid Equivalent/100 g and 129.2 to 219.12 mg QE/100 g, respectively [13]. *M. nigra* L. contains higher amounts of bioactive elements than *M. alba* L., largely due to anthocyanins [19].

Chitosan (Cht) is a natural polymer obtained by deacetylating chitin, and it is widely used in fields such as environmental protection, nutrition, biotechnology, and medical care [20]. Cht stands out as an exceptional material for wound dressings because of its compatibility with living tissues, antibacterial features, and natural blood-clotting abilities. Chitosan nanoparticles for drug delivery without injections have broad uses in managing diseases like cancer, digestive issues, and respiratory problems [21].

The skin, together with nails, hair, and glands, constitutes the integumentary system, serving as a shield that divides the body’s interior from the outside world. As the largest organ, skin covers about 22 square feet [22].

Skin is vital for body protection, since most human infections enter via this layer. Thus, it is seen as the first defense mechanism and main barrier [23]. Many skin conditions, including cancers, herpes, eczema, fungal issues, itching, athlete’s foot, color changes, aging signs, bug bites, psoriasis, injuries, acne, and various wound infections, create major hurdles in healthcare [24].

In efforts to treat these skin problems, remedies from natural sources like animals and plants—known as natural products—have become more popular. Despite advances in science and technology, using natural bioproducts optimally for skin disorder treatment has become essential in fighting infections [25]. This trend stems from rising interest in herbal options, their low cost, and greater recognition of side effects from conventional drugs. Lately, medical experts have noted more cases of hard-to-heal wounds and burns, highlighting the need for new approaches [26].

Antimicrobial/hygienic textiles’ fabrication has great potential for treating skin infections and promoting tissue regeneration, especially with their loading with bioactive nanomaterials and natural derivatives [22,25]. The usage of plant-mediated nanometals (e.g., AgNPs, SeNPs, ZnONPs, …) for producing/finishing antimicrobial textiles was advocated to generate highly effectual fabrics with skin-protectant potentialities for wound/burn infections, UV protection, anti-aging properties, and skin regeneration [22,23,24,25,26]. Furthermore, several biopolymers, nanoforms, and nanocomposites were effectually employed for functional textile finishing to provide promising characteristics for fabrics, including antimicrobial properties, healing stimulation, increased strength, and skin protection [22,23,25].

Polysaccharides and biopolymers have great potential in nanotechnology sectors; such polymeric materials (e.g., from marine and plant sources) can assist in nanometals’ biosynthesis and functionality through reduction, capping, stabilizing, and synergizing their bioactivities in antioxidant, anticancer, or antimicrobial nanocomposites [27,28,29,30,31,32]. The applications of Cht and its nanoforms/composites as antimicrobial agents has had promising success. Cht (with its positive surface charges) can attach and suppress/interact with microbes (mostly with negative structures) and interfere with membrane synthesis, proteins’ functions, and DNA/RNA comportments, which leads to microbial cell destructions and death [20,21,29,30,31]. Constructions of bioactive Cht-based nanocomposites were documented to possess remarkable biocidal actions toward cancer lines and pathogenic microorganisms [29,30,31,32]. However, compared to former studies that evaluated Cht-based nanocomposites with other plant polysaccharides and nanometals [31,32], these promising results promote the search for further innovative applications of Cht-based nanocomposites with additional bioactivities and applicability.

Therefore, the objectives of this study were the innovative extraction of polysaccharides from black mulberry (MB), biosynthesizing SeNPs with MB, constructing inventive bioactive nanocomposites comprising nanochitosan (Cht) and MB-SeNPs, developing antimicrobial fabrics loaded with these nanocomposites, and assessing their antimicrobial effects on skin-related microbial pathogens.

## 2. Materials and Methods

### 2.1. Materials

All experimental supplies and chemicals were of verified analytical purity; the ethanol, Na_2_SeO_3_ (≥98%), methanol, nutrient agar (NA), nutrient broth (NB), yeast malt (YM) agar and broth, and chitosan (“Medium molecular weight; deacetylation >75%, Product Number: 448877”) were attained from Sigma Aldrich in St. Louis, MO, USA. Double-distilled water (DDW) was utilized for extracting mucilage and preparing solutions.

### 2.2. Extraction of Black mulberry (Morus nigra L.) Polysaccharide

Fresh organic black mulberry fruits (*Morus nigra* Linn.) were obtained from a certified vendor in Jeddah, Saudi Arabia. The fruits were selected, washed multiple times with DDW, and dried in an air oven at 47 °C for 44–50 h. The dried material was ground mechanically, and the powder was mixed in 15 times its volume of DDW for 24 h using a shaker (KS-4000 I control, IKA, Staufen, Germany), followed by filtration through muslin cloth. The black mulberry (MB) polysaccharide was precipitated using absolute ethanol at twice the volume of the aqueous extract [27]. The polysaccharides were collected via centrifugation (9350× *g* for 28 min) with a cooling centrifuge (SIGMA 2-16KL; Sigma Lab. GmbH, Osterode am Harz, Germany) and dried in an oven at about 42 °C for 48 h [28]. The solid MB was ground and kept in a desiccator.

### 2.3. Phytosynthesis of SeNPs with MB

First, a 10 mM aqueous solution of sodium selenite (Na_2_SeO_3_) was made in DDW. Then, 10 mL of MB solution (1%, *w/v* in DDW) was combined with an equal volume of Na_2_SeO_3_ and stirred (610× *g*) using a magnetic stirrer (AREX-6; VELP Scientific Srl., Usmate, Italy) for 60 min at room temperature (25 ± 2 °C) [29]. A few drops (~0.2 mL) of ascorbic acid (0.5% concentration, *w/v*) were added to start the reduction. The formation of SeNPs was evident by the color shift to a brownish-orange hue. The MB-SeNPs were separated by centrifugation at 12,300× *g* for 33 min. To purify the SeNPs, the MB-SeNPs were washed three times with DDW and twice with ethanol, followed by centrifugation [30]. The collected MB-SeNPs and SeNPs were rinsed with DDW, frozen overnight, freeze-dried, and analyzed.

### 2.4. Preparation and Loading of Cht

A 1% (*w/v*) chitosan solution was prepared via dissolving in acetic acid solution (1.5%, *v/v*, dilution). Sodium tripolyphosphate solution (TPP, 0.1%, *w/v*) was slowly added dropwise to a 1:1 mixture of chitosan and MB-SeNP solutions under constant magnetic stirring, then centrifuged, washed with DDW, recentrifuged, and dried [30]. Various ratios of Cht/MB-SeNPs were tested to find the best mix: a trial (F-1) at a 1:2 ratio of Cht:MB-SeNPs, a trial (F-2) at 1:1, and a trial (F-3) at 2:1. The usage of the selected mixing ratios was based on former studies that intermixed chitosan with other polysaccharides [31,32].

### 2.5. Characterization of Nanomaterials/Nanocomposites

#### 2.5.1. FTIR (“Fourier-Transform Infrared Spectroscopy”)

FTIR was used to identify chemical bonds, functional groups, and interactions in the mucilage and other compounds via their infrared absorption patterns. Transmission was measured from 4000 to 450 cm^−1^, after mixing dried powders with KBr and subjecting them to analyses using “FT-IR-360, Fourier transform infrared spectroscopy, JASCO, Tokyo, Japan”.

#### 2.5.2. Zeta (ζ) Potential and Particles’ Size

The dimensions, dispersion, and surface charges of Cht, MB-synthesized SeNPs, and their composites (Cht/MB-SeNPs; F-1, F-2, F-3) were assessed using dynamic light scattering (DLS) with a Zetasizer (Zeta plus, Brookhaven, NY, USA). SeNPs’ size and distribution stability were checked over 72 h post-synthesis. The crystalline nature of the MB-SeNPs was also examined with X-ray diffractometry (XRD; Siemens, D500, Munich, Germany).

#### 2.5.3. Scanning Electron Microscopy (SEM)

SEM (JEOL, JSM IT100, Tokyo, Japan) was applied to evaluate the size, form, and surface features of the Cht/MB-SeNP nanocomposites after sample mounting and coating with gold/palladium.

#### 2.5.4. Transmission Electron Microscopy (TEM)

The distribution, dimensions, and shape of the MB-photosynthesized SeNPs were analyzed using TEM (JEOL, JEM-2100, Tokyo, Japan) at a 200 kV acceleration voltage.

### 2.6. Antimicrobial Testing Assays

The antimicrobial effects of the Cht, MB-synthesized SeNPs, and their composites were assessed against skin pathogens; *Staphylococcus aureus* (ATCC 6538) was screened as the bacterial model and *Candida albicans* (ATCC 24433) was the yeast model. The bacteria and yeast were tested qualitatively and quantitatively to emphasize the nanomaterials’ activity against prokaryotes and eukaryotes, respectively [31]. *S. aureus* was grown in NB and NA, while *C. albicans* used YM, incubated aerobically at 37 °C.

#### 2.6.1. Qualitative Assay

Clear zones without growth (IZ) indicated antimicrobial strength in disk diffusion [31]. Diluted solutions (0.1%, *w/v*) of materials were loaded on filter disks. After spreading microbes on agar, disks were placed and incubated for 24 h at 37 °C. Average IZ diameters were recorded [32].

#### 2.6.2. Minimum Inhibitory Concentration (MIC) Quantitative Assay

MICs of MB-SeNPs, F-1, F-2, and F-3 against pathogens were found in broth media dosed from 5 to 100 µg/mL, using a macro-dilution technique [31]. Tubes of broth media (NB and YM) were amended with nanomaterial solution at specified concentrations; then each tube received 200 µL of 10^6^ CFU/mL from 24 h cultures in 5 mL amended broth, shaken at 175× *g* for 24 h at 37 °C. Samples (100 μL) from each treatment were plated onto fresh agar plates and incubated; the lowest concentrations that led to growth-free plates were defined the MICs [33].

#### 2.6.3. SEM Imaging of Challenged Microorganisms

Antibacterial effects of Cht/MB-SeNPs on *S. aureus* and *C. albicans* were observed via SEM after incubating in MIC-amended broth for 5 and 10 h at 37 °C. Cells were centrifuged, mounted, dehydrated with an ethanol series, coated with gold/palladium, and scanned for damaged structures.

### 2.7. Antimicrobial Textile Preparation

Plain-weave cotton gauze (86.21 g/m^2^, scoured) from Misr Weaving/Spinning Co., Nile Delta, Egypt, was sterilized and treated with solutions containing 1.0% of MB-SeNPs or Cht/MB-SeNPs (e.g., F-2). Using the “Pad–Dry–Cure” method, adapted from earlier works [30,31]; fabric was soaked in nanomaterial solutions for 100 min at room temperature with gentle stirring, padded to 100% wet pickup, dried for 120 min in forced air, and cured for 15 min at 80 °C. For testing, fabrics were cut into ~2.0 cm^2^ pieces and tested for IZs against microbes on inoculated agar (e.g., NA for *S. aureus* and YM for *C. albicans*), based on AATCC-147 (“the Antimicrobial Textile Test; The American Association of Textile Chemists and Colorists”), after incubation for 24–36 h at 37 °C [30,31]. Triplicate IZ diameters were averaged around fabric pieces after incubation.

The durability of textiles after washing was assessed using the standard AATCC-100 test method [34] for evaluating antimicrobial durability in textiles after washing with 2 successive cycles, which quantifies the antimicrobial activity of a treated fabric over a 24 h period. Antimicrobial activity is expressed as percentages, by comparing microbial counts after each wash cycle with counts in unwashed samples.

### 2.8. Statistical Analysis

Results were expressed as means ± SDs from triplicate tests using SPSS V-11.5 (Chicago, IL, USA). Differences were analyzed with one-way ANOVA, significant at *p* ≤ 0.05 [30].

## 3. Results and Discussion

### 3.1. Preparation of Cht/MB-SeNP Nanocomposites

#### 3.1.1. Optical Observation

**Figure 1:**
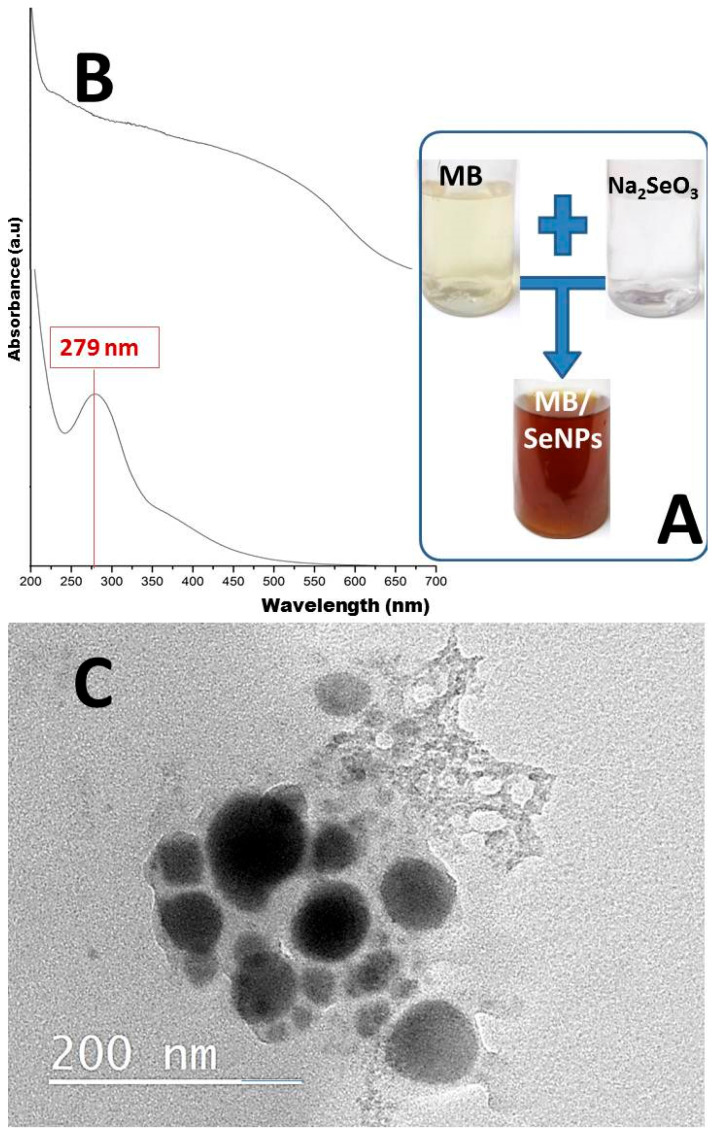
Optical observation (**A**), UV spectral analysis (**B**) of MB solution (upper) and MB-SeNPs (lower curve), and transmission microscopy imaging (**C**) of SeNPs biosynthesized with mulberry polysaccharides.

MB facilitated the bioreduction of Na_2_SeO_3_ to SeNPs, shown by the color change from pale yellow to deep brownish-orange in 60 min (Figure 1A), confirming SeNP formation [33]. As noted earlier, Se nanomaterials’ unique traits depend on size and structure, adjustable via synthesis parameters [9]. A peak near 279 nm relates to SeNP crystal inter-chain links (Figure 1B, lower curve), whereas the MB spectrum (upper curve) does not indicate a distinctive peak of absorbance. Thus, a low-energy peak at longer wavelengths reveals inter-chain details and crystallinity [9,32].

TEM images (Figure 1C) display MB-synthesized SeNPs as spherical, evenly distributed, and sized 16.76–124.32 nm, with little clumping. SeNP variations are tied to the extract reduction strength. The TEM results (Figure 1C) highlight MB’s role in reducing and stabilizing SeNPs. Key MB components for reduction include anthocyanins (e.g., cyanidin-3-glucoside, cyanidin-3-rutinoside), polyphenols, and flavonoids (rutin, isoquercitrin, catechin, dihydroquercetin, hesperidin, neohesperidin, quercetin, naringenin, petunidin 3-glucoside, chlorogenic acid, cyanidin-3-O-glucoside), granting strong antioxidative and reductive abilities [35,36,37,38].

#### 3.1.2. FTIR Analysis

**Figure 2:**
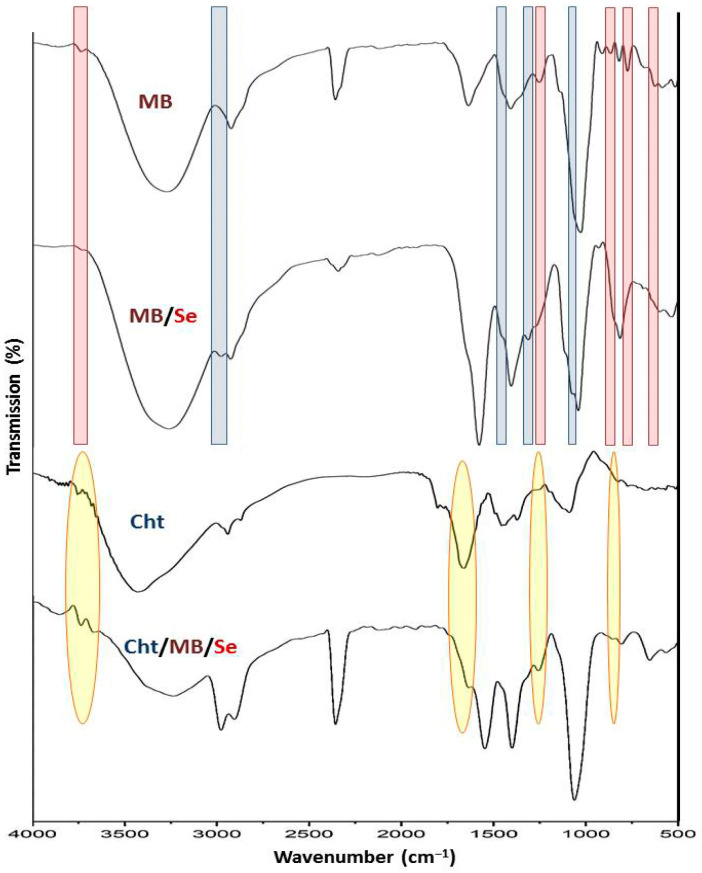
FTIR patterns for the extract of mulberry (MB) polysaccharides, phytosynthesized selenium nanoparticles with the MB polysaccharides (MB/Se), chitosan (Cht), and the combined nanocomposites (Cht/MB/Se).

Figure 2 shows FTIR spectra for MB, Cht, MB-SeNPs, and Cht/MB-SeNPs. MB’s lyophilized samples were scanned from 4000 to 500 cm^−1^ (Figure 2—MB), with peaks at 3220–3450, 2917, 2382, 1627, 1425, 1255, 1114, 924, 885, 762, and 615 cm^−1^. The 3220–3450 cm^−1^ band suggests O-H from carbs or ketones/carboxylic acids [37,38,39]. The 2917 cm^−1^ band indicates C-H stretch, and 1627 cm^−1^ likely indicates C=O stretch [39,40,41].

SeNP bioreduction and MB interactions appear in spectral differences between the plain MB (Figure 2—MB) and MB-SeNPs (Figure 2—MB/Se). New or intensified bands (blue highlights) at ~1105, 1285, 1480 cm^−1^, and 2920–3050 cm^−1^ show new bonds in synthesis. Diminished or vanished bands (red highlights) at ~640, 765, 870, 1252, and 3745 cm^−1^ confirm bond involvement or disruption with SeNPs [42].

For Cht (Figure 2—Cht), bands include ~3430 cm^−1^ for N-H/O-H hydrogen bonds, 2927/2859 cm^−1^ for C-H stretch, 1683 cm^−1^ for N-H bend, 1371 cm^−1^ for O stretch, and 1145 cm^−1^ for C-O-C [43,44].

The composite spectrum (Figure 2—MB/Cht/SeNPs) retains bonds from components, mainly indicating physical bonds [45]. Yellow highlights show Cht-transferred bands in the nanocomposites at ~868 cm^−1^, 1258 cm^−1^, 1635 cm^−1^, and 3650–3820 cm^−1^, with others are from MB-SeNPs.

#### 3.1.3. Ps Distribution and NP Charge

Table 1 details ratios, particle size (Ps) distribution, and ζ potential for Cht, MB-SeNPs, and Cht/MB-SeNPs. MB produced SeNPs with an average particle size of 46.19 nm. SeNPs had a −27.93 mV ζ-potential, while that of the F-3 nanocomposites averaged 266.16 nm (49.77–692.25 nm) with +34.37 mV. The SeNPs’ stability showed minor changes over 72 h.

The F-1 nanocomposites had a wider Ps (59.23–678.39 nm) and a 239.88 nm average, reflecting integration. F-2 (equal ratio) had the smallest average (212.42 nm). ζ potentials indicated high stability, matching reports on Cht and bio-SeNPs composites [46]. MB-SeNP/Cht interactions yielded varied ζ, negative for higher MB-SeNP levels (F-1, F-2) and positive for Cht-dominant F-3. This aligns with prior studies using polysaccharides/mucilage with Cht for nanometals [32,47,48], which could be considered in prospective investigations of further biopolymer nanocomposites.

#### 3.1.4. SEM Imaging

**Figure 3:**
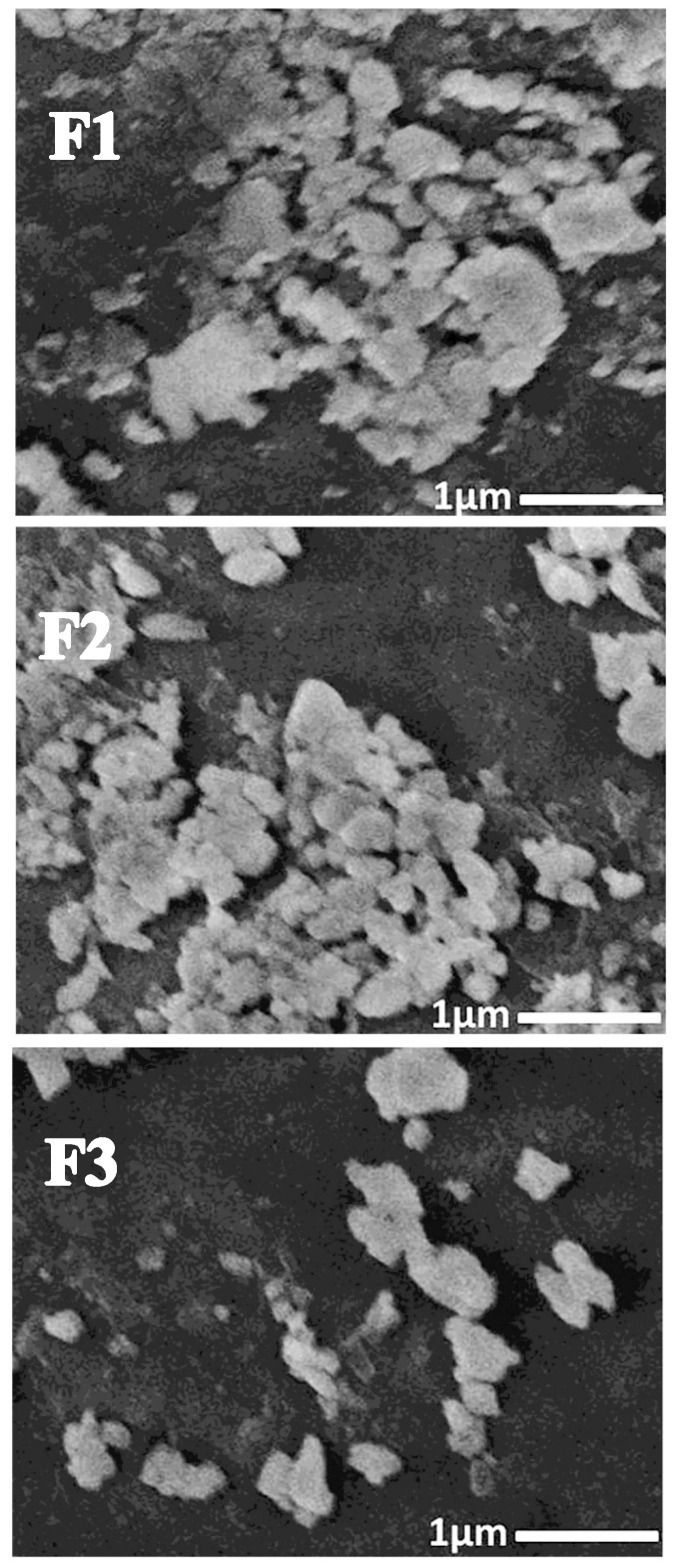
Scanning microscopy micrographs of different nanocomposites fabricated from nanochitosan and mulberry mucilage-mediated SeNPs. The nanocomposites were constructed from nanochitosan and MB-SeNPs at ratios of 2:1 (F1), 1:1 (F2), and 1:2 (F3).

SEM of nanochitosan revealed particle distribution and form [32,49]. Figure 3 shows Cht/MB-SeNP nanocomposites as semi-spherical, with sizes matching those detected by DLS (Table 1). The best blend was F-2, then F-1 and F-3. Cht had a +38.53 mV ζ, with SEM confirming dispersion, uniformity, and sphericity [48]. The SEM imaging of nanocomposites was used rather than TEM to emphasize the apparent topography and structure of nanoparticles, which gave more reliability about their dispersion and uniformity [43,44,48,49].

Nanocomposites’ morphology and charge can affect their antimicrobial bioactivities, as tiny positive particles aid microbial attachment, as well as encapsulated agents [30,49]. Synergistic actions of biopolymer NCs enhance safety for nanometals in matrices [29,30], crucial for antimicrobial potency and tissue safety.

### 3.2. Antibacterial Assay

#### 3.2.1. Qualitative and Quantitative Assays

MB-SeNPs’ and Cht/MB-SeNPs’ effects on skin pathogen models (*C. albicans* yeast and *S. aureus* bacteria) were quantified by MICs and qualified by ZOIs (Table 2). F-2 showed superior activity with larger ZOIs and lower MICs. NP activity correlates with size [30,47]; smaller NPs increase surface contact with membranes, boosting permeability and cellular entry [9,48]. The nanomaterials attained herein, with their minute particles and elevated bioactivities, could advocate investigations of further nanocomposites with diverse sizes as antimicrobial nanomaterials. The former relevant compositing of Cht with AgNPs that were synthesized with royal jelly generated potent antifungal nanoconjugates for suppressing *C. albicans*, which supports the current results in this study [49].

#### 3.2.2. Antibacterial Elucidation via SEM

**Figure 4:**
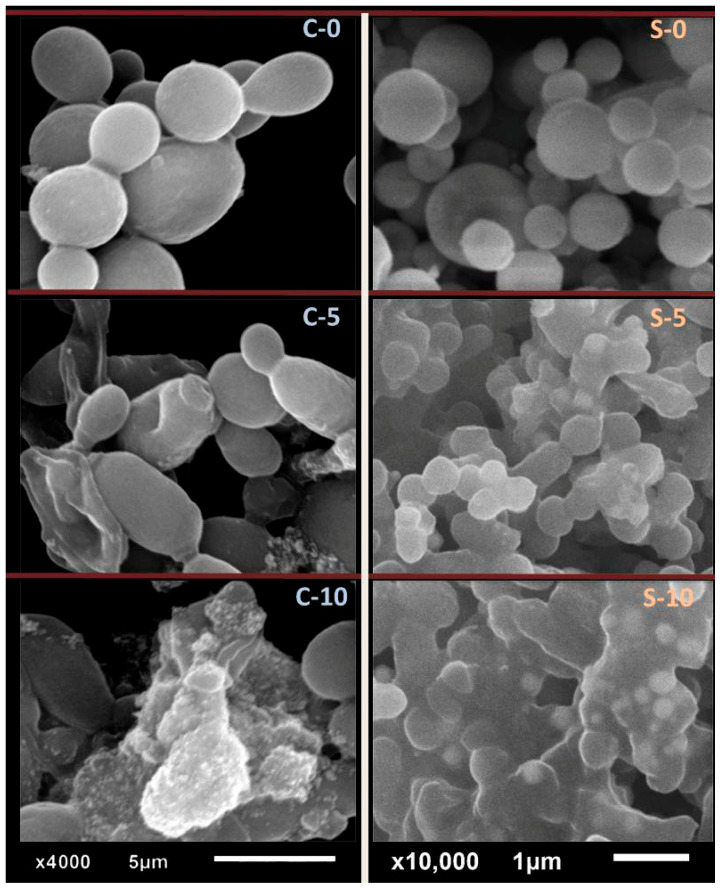
Scanning microscopy imaging of *Candida albicans* and *Staphylococcus aureus* cells exposed to nanocomposites of nanochitosan and mulberry polysaccharide-mediated SeNPs for different exposure times. Letters in the figure indicate microbes; C stands for *Candida albicans* and S for *Staphylococcus aureus*, whereas numbers (0, 5, and 10) indicate the exposure time in hours.

In Figure 4, the SEM shows effects of Cht/MB-SeNPs (F-2) on *S. aureus* and *C. albicans*. Initially, *C. albicans* (C-0) appeared normal with smooth walls. After 5 h (C-5), cells swelled with Se-NPs attached. By 10 h (C-10), lysis and debris mixed with NPs were present.

For *S. aureus* (S-0), cells were healthy. After 5 h (S-5), swelling and NP attachment appeared; by 10 h (S-10), full distortion and lysis were present. Effects stemmed from combined MB/Cht and SeNP mechanisms. The main antimicrobial mechanism of the prepared materials against bacteria and yeast involved the synergized actions of the components (particularly AgNPs and Cht) [31,49]. Cht has positively charged particles that facilitate attachment onto microbial surfaces, entrance from outer membranes, and interference with and suppression of cellular biosystems, in addition to capability to encapsulate further active molecules within its particles [31,32,43,44]. AgNPs’ antimicrobial action mainly involves high penetration into cells, reactive oxygen (ROS) species generation, and bio-toxicity toward cellular organelles/systems [42,48,49].

### 3.3. Antimicrobial Textile Preparation

**Figure 5:**
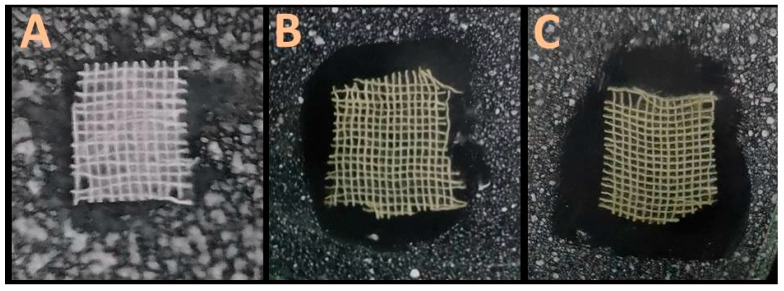
Examples of antimicrobial activity from cotton textiles treated with 1% mulberry-mediated selenium nanoparticles (**B**) and their composites with nanochitosan (**C**) compared to that of control textiles (**A**) against antibiotic-resistant strain of *Staphylococcus aureus*.

Table 3 and Figure 5 show the antimicrobial effects of Cht/MB-SeNP-loaded fabrics on skin pathogens. Cht/MB-SeNP fabrics outperformed MB-SeNPs alone, with *C. albicans* being more sensitive than *S. aureus*, and antibiotic-sensitive strains were more affected than resistant ones.

Cht/MB-SeNPs showed strong activity against infections, including resistant strains, due to multiple agents in MB and Cht, as well as Cht’s adhesion to cells. Combined actions suppress microbes effectively [29,30,49]. Interestingly, the loaded fabrics could preserve their antimicrobial activity after two successive washing cycles, with 93.41 and 84.28% reduction capacity against *C.albicans* and 92.64 and 80.81% against *S. aureus*, compared to unwashed samples, after first and second wash cycles, respectively. This indicates the applicability, durability, and potentiality of loaded textiles for upholding nanomaterials, even after multiple washing cycles [22,30,31,47,49]. Durability arises from synergistic cross-linking with fibers, reducing wash-off [31,47].

The antimicrobial impacts of treated fabric were lower than those of plain Cht, MB-SeNPs, and Cht/MB-SeNPs against challenged microbes, which could be attributed to nanomaterials’ attachment to/intermixing with textile fibers and because many challenges can appear during fabric processing [30,31]. Beyond combined efficacy, gradual release from fibers benefits prolonged contact with infected skin. These fabrics could produce bandages, gloves, and skin protectors. Promisingly, most of materials used herein (e.g., Cht and MB) could be considered as GRAS (“Generally Recognized As Safe”), whereas the biosafety of biogenic synthesized SeNPs was warranted in many investigations [8,9,32,33], which advocates their applicability and compatibility in most human-related disciplines. Green approaches for nanomaterial/nanocomposite biosynthesis provide additional biosafety characteristics to these compounds and support their non-harmful usage for human purposes [5,11,24].

Further improvements to the illustrated process can be suggested, including the examination/application of additional biopolymer nanocomposites with diverse functionalities, the extensive evaluation of finished textiles using modern methods, and the evaluation of fabric biosafety and allergenic reactions using animal models.

## 4. Conclusions

The novel biosynthesis of MB-mediated SeNPs and Cht nanocomposites succeeded in creating advanced antimicrobial and skin-protective agents. The MB was extracted from mulberry fruits and directly employed for SeNP biosynthesis using a facile method, before direct conjugation with Cht to construct Cht/MB-SeNP nanocomposites, with mean sizes of 212.42–266.16 nm. The nanoparticles displayed excellent antibacterial traits and favorable physical–chemical properties. Observed inhibitions confirmed the Cht/MB-SeNPs’ effectiveness against *S. aureus* and *C. albicans*. The 1:1 Cht/MB-SeNP mixture (F-2) proved most effective, with inhibition zones of 26.3 mm against *S. aureus* and 27.1 mm against *C. albicans*. The nanocomposites caused major microbial cell damage; loaded fabrics also exhibited strong antimicrobial effects. Cotton fabrics’ loading with Cht/MB-SeNP nanocomposites could produce durable antimicrobial textiles that could prohibit microbial growth and preserve their antimicrobial actions after two washing cycles. This work originally offers key contributions to antimicrobial innovation, highlighting biosynthesized nanocomposites as eco-friendly and potent options against skin threats. Future studies and uses promise advancements in safe treatments for skin issues. Further tests/methods could be suggested, such as absorbent capacity, permeability (air/moisture), tensile, elongation, tear, bending, EDS-TEM, and ED-TEM analyses, to validate crystallinity and atomic composition for prospective investigations.

## Figures and Tables

**Table 1:** Size distribution and zeta (ζ) potential of fabricated nanochitosan (Cht), biosynthesized selenium nanoparticles with MB (MB-SeNPs), and their nanocomposites (Cht/MB-SeNPs) *.

Biopolymer Composition	Chitosan–Mucilage Ratio	Particle Size Range (nm)	Particle Size Mean (nm)	Zeta Potential (mV)
Chitosan	1:0	ND	ND	+38.53 ± 1.41
MB	0:1	ND	ND	−26.37 ± 1.09
MB-SeNPs	0:1	16.76–124.32	46.19	−27.93 ± 0.62
F-1	1:2	59.23–678.39	239.88	−24.11 ± 0.73
F-2	1:1	39.16–720.83	212.42	−16.88 ± 0.84
F-3	2:1	49.77–692.25	266.16	+34.37 ± 1.36

* ND: not detected; F-1, F-2, and F-3: the nanocomposites formulated from nanochitosan and mulberry-biosynthesized SeNPs at ratios of 2:1 (F1), 1:1 (F2), and 1:2 (F3).

**Table 2:** Antimicrobial activities of synthesized nanocomposites against skin pathogens measured as zones of inhibition (ZOIs, mm) and minimal inhibitory concentrations (MICs, μg/mL).

Nanocomposites	Skin Pathogens			
	*Staphylococcus aureus*		*Candida albicans*	
	ZOI (mm) *	MIC (µg/mL)	ZOI	MIC
MB-SeNPs	18.5 ± 1.4 ^a^	32.5	19.1 ± 1.5 ^a^	35.0
F-1	22.4 ± 1.7 ^b^	27.5	24.2 ± 1.9 ^a^	30.0
F-2	26.3 ± 2.2 ^c^	22.5	27.1± 2.5 ^b^	20.0
F-3	24.8 ± 1.8 ^c^	25.0	25.2 ± 1.7 ^c^	25.0

* The zones are triplicate means (mm), including assay disks’ diameters; dissimilar superscript letters within a column indicate differences with significance at *p* < 0.05.

**Table 3:** Antimicrobial activity of cotton textiles loaded with 1% nanochitosan (Cht), mulberry polysaccharide-mediated selenium nanoparticles (MB-SeNPs), and their nanocomposites against antibiotic-resistant strains from *Candida albicans* and *Staphylococcus aureus*.

Antimicrobial Agent	Zone of Inhibition Toward Skin Pathogens *	
	*Candida albicans*	*Staphylococcus aureus*
Control (1.0% acetic)	Not Detected	Not Detected
Cht	3.3 ± 0.8 ^a^	2.9 ± 0.6 ^a^
MB-SeNPs	4.5 ± 1.1 ^b^	3.3 ± 0.7 ^b^
Cht/MB-SeNPs	6.2 ± 1.6 ^c^	3.7 ± 0.8 ^b^

* The zones are triplicate means (mm), excluding the textile pieces’ diameters; dissimilar superscript letters within a column indicate differences with significance at *p* < 0.05.

## Data Availability

The original contributions presented in this study are included in the article. Further inquiries can be directed to the corresponding author.

## Abbreviations

The following abbreviations are used in this manuscript:

MB	Black mulberry polysaccharide
Cht	Chitosan
SeNPs	Selenium nanoparticles
PBS	Phosphate-buffered saline
TEM	Transmission electron microscopy
SEM	Scanning electron microscopy
DLS	Dynamic light scattering
FTIR	Fourier-transform infrared spectroscopy
ζ	Zeta potential
DDW	Double-distilled water
ZOI	Zone of inhibition
MIC	Minimum inhibitory concentration
RT	Room temperature
